# A hybrid type I, multi-center randomized controlled trial to study the implementation of a method for Sustained cord circulation And VEntilation (the SAVE-method) of late preterm and term neonates: a study protocol

**DOI:** 10.1186/s12884-022-04915-5

**Published:** 2022-07-26

**Authors:** Katarina Ekelöf, Elisabeth Sæther, Anna Santesson, Maria Wilander, Katarina Patriksson, Susanne Hesselman, Li Thies-Lagergren, Heike Rabe, Ola Andersson

**Affiliations:** 1 Department of Clinical Sciences Lund, Pediatrics/Neonatology, Lund University, Lund, Sweden; 2 Department of Women, Children and Adolescents, Ålesund Hospital, Møre and Romsdal Hospital Trust, Ålesund, Norway; 3 Department of Clinical Sciences Lund, Child and Adolescent Psychiatry, Lund University, Lund, Sweden; 4 Department of Pediatrics, Hospital of Halland, Halmstad, Halmstad, Sweden; 5 Department of Health Sciences, University West, Trollhättan, Sweden; 6 Division of Pediatrics, NU-Hospital Group, Trollhättan, Sweden; 7 Department of Women’s and Children’s Health, Uppsala University, Uppsala, Sweden; 8 Center for Clinical Research, Falun, Sweden; 9 Department of Midwifery Research - Reproductive, Perinatal and Sexual Health, Lund University, Lund, Sweden; 10 Brighton and Sussex Medical School, University of Sussex, Brighton, UK; 11 Department of Neonatology, Sussex University Hospitals NHS Trust, Brighton, UK; 12 Department of Neonatology, Skåne University Hospital, Malmö/Lund, Sweden

**Keywords:** Intact cord resuscitation, Placental transfusion, Implementation, Delayed cord clamping practice, Optimal cord management

## Abstract

**Background:**

An intact umbilical cord allows the physiological transfusion of blood from the placenta to the neonate, which reduces infant iron deficiency and is associated with improved development during early childhood. The implementation of delayed cord clamping practice varies depending on mode of delivery, as well as gestational age and neonatal compromise. Emerging evidence shows that infants requiring resuscitation would benefit if respiratory support were provided with the umbilical cord intact. Common barriers to providing intact cord resuscitation is the availability of neonatal resuscitation equipment close to the mother, organizational readiness for change as well as attitudes and beliefs about placental transfusion within the multidisciplinary team. Hence, clinical evaluations of cord clamping practice should include implementation outcomes in order to develop strategies for optimal cord management practice.

**Methods:**

The Sustained cord circulation And Ventilation (SAVE) study is a hybrid type I randomized controlled study combining the evaluation of clinical outcomes with implementation and health service outcomes. In phase I of the study, a method for providing in-bed intact cord resuscitation was developed, in phase II of the study the intervention was adapted to be used in multiple settings. In phase III of the study, a full-scale multicenter study will be initiated with concurrent evaluation of clinical, implementation and health service outcomes. Clinical data on neonatal outcomes will be recorded at the labor and neonatal units. Implementation outcomes will be collected from electronic surveys sent to parents as well as staff and managers within the birth and neonatal units. Descriptive and comparative statistics and regression modelling will be used for analysis. Quantitative data will be supplemented by qualitative methods using a thematic analysis with an inductive approach.

**Discussion:**

The SAVE study enables the safe development and evaluation of a method for intact cord resuscitation in a multicenter trial. The study identifies barriers and facilitators for intact cord resuscitation. The knowledge provided from the study will be of benefit for the development of cord clamping practice in different challenging clinical settings and provide evidence for development of clinical guidelines regarding optimal cord clamping.

**Trial registration:**

Clinicaltrials.gov, NCT04070560. Registered 28 August 2019.

**Supplementary Information:**

The online version contains supplementary material available at 10.1186/s12884-022-04915-5.

## Background

At birth, circulation between the placenta and the neonate continues for several minutes. Waiting to clamp the umbilical cord promotes the physiological transfusion of placental blood to the neonate and thereby enhances postpartum adaptation [1, 2]. Enabling the placental transfusion reduces adverse neonatal outcomes and improves development during early childhood [2].

A systematic review by Anton [3] of 18 publications, which included quality improvement projects on delayed cord clamping (CC), identified common barriers to implementing optimal cord management methods. Barriers identified were concerns regarding neonates needing resuscitation, as well as births complicated by cesarean section or postpartum hemorrhage [3].

The implementation of delayed CC practice varies between countries, and international and national guidelines differ depending on mode of delivery and complications at birth [4]. Hence, more studies of specific clinical situations and different implementation settings are needed.

In 2006, most labor departments in Sweden practiced immediate cord clamping (< 30 s) for all neonates. In 2008, a set of CC recommendations was published as the Swedish standard [5]. This led to the gradual adoption of a new standard and to the practice of implementing CC after 2–3 min. However, implementing new evidence-based knowledge and new interventions takes time, and different interventions have different determinants for implementation [6]. Concerning vigorous neonates, a national telephone survey in 2015 reported that midwives in Sweden clamped the cord after cessation of pulsations [7]. An observational study on 904 vaginal births in Sweden 2018 showed an average CC time of 6 min [8].

When a neonate in need of resuscitation is born, immediate clamping and cutting of the umbilical cord is still recommended according to several guidelines (see an overview in Supplement 1). Pilot studies have shown that resuscitation with an intact cord is a feasible practice for term non-vigorous vaginally born neonates, and data have shown there are long-term benefits during infancy [9–11]. Yet, larger randomized control trials are needed to supply reliable evidence regarding the appropriate steps to be taken during intact cord resuscitation (ICR) [3, 12].

The availability of equipment for resuscitation is one limiting factor for providing ICR. The local setting, routines, and position of resuscitation equipment differ between hospitals. In most hospitals, resuscitation equipment is placed at some distance from the delivery bed, or even in a separate room. Mobile platforms (e.g. LifeStart™, Concord™, BabySaver Tray™) have been developed with the intent of enabling stabilization of preterms with an intact cord [13, 14]. Studies of the feasibility and acceptability of this equipment have concluded that use of this approach is beneficial [9, 15–17].

As cost and availability of the mobile platform are important factors, particularly in labor departments in medium- and low-income countries, we developed a method to provide ICR for near-term and term neonates in the mother's bed (“in-bed” ICR) in a pilot study in Nepal. The study demonstrated improved early neonatal outcomes such as oxygenation after birth and Apgar score [18].

Implementing new methods successfully in clinical routines requires addressing contextual factors such as attitudes (acceptability), professional behavior (adoption) and the health care service (efficiency and safety). These factors will also impact the clinical outcomes [19]. The resuscitation setting is a complex context with different professionals involved. Well-functioning teamwork among the members of a multidisciplinary team (MDT) is fundamental to ensuring the success of delayed cord clamping practice [3]. Hence, it is important to learn about the individual attitudes and views on employing delayed CC as expressed by team members with different backgrounds and professions. Addressing barriers and facilitators to ICR during the clinical evaluation process will identify essential factors for implementation success [3].

The objective of the study is to:

1. Develop and evaluate a method for “in-bed” ICR in late preterm and term infants
2. Determine and examine implementation factors for “in-bed” ICR
3. Evaluate implementation outcomes (appropriateness, acceptability, adoption, fidelity, feasibility) and health service outcomes for “in-bed” ICR.

**Fig. 1:**
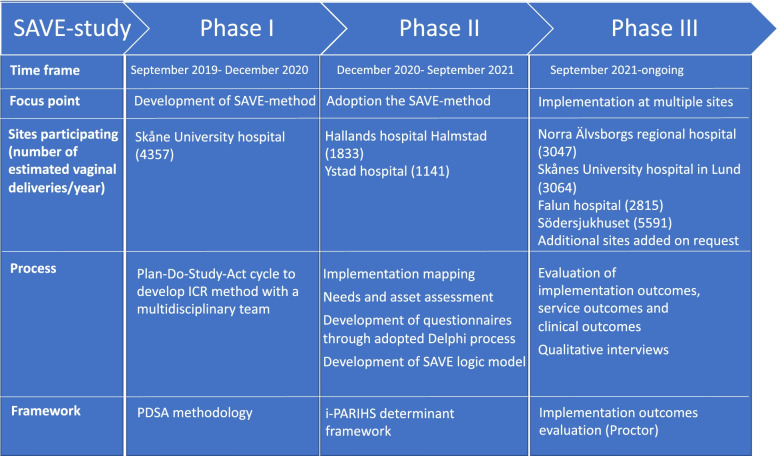
Overview of the SAVE-study from the pilot study in phase I to starting up additional sites and adopting the method in phase II and the implementation in the multicenter study in phase III. SAVE-method – Sustained cord circulation And VEntilation. MDT – multidisciplinary team. ICR – intact cord resuscitation. i-PARIHS – integrated-Promoting Action on Research Implementation in Health Services determinant framework. PDSA – Plan-Do-Study-Act

We report the development of a method for “in-bed” ICR (the SAVE-method) and the design of the prospective evaluation in a randomized controlled hybrid type 1 multicenter trial in level II and III hospitals in Sweden. An overview of the different phases of the study is shown in Fig. 1. The SAVE (Sustained cord circulation And Ventilation) study will contribute to knowledge of significant aspects of implementation of new guidelines for cord clamping for neonates requiring resuscitation.

## Study overview

### Study design

The SAVE study is a hybrid type 1 randomized controlled multicenter trial [20].

### Trial registration

Clinicaltrials.gov, NCT04070560. Registered 28 August 2019, https://clinicaltrials.gov/ct2/show/NCT04070560

### Study setting

Seven level II and III hospitals in Sweden are included in the study. Start-up was initiated in different phases from initial pilot study (phase I) to additional sites being added on to end in a multicenter trial (phase III) see description below and Fig. 1.

### Phase I: Development of the SAVE-method

We chose to use a stepwise Plan-Do-Study-Act (PDSA) approach containing protocol development, targeted education, multidisciplinary team involvement and constructive feedback loops [21] as identified by Anton et al. as factors to be considered in evaluating delayed CC-implementation [3].

First, the gaps in knowledge and evidence for CC practice in term infants requiring resuscitation were identified. A hypothesis and a project plan of the clinical evaluation of ICR were developed. The study setting was defined and support from management teams (head of department, unit managers at the labor department and neonatal unit) as well as from medical responsible obstetricians and neonatologists was ensured. An MDT was formed at the Skåne University hospital in Malmö and was given responsibility for developing a method for “in-bed” ICR. The MDT consisted of midwives, obstetricians, an auxiliary nurse, neonatal nurses, neonatologists, as well as unit managers. The MDT reference group identified potential challenges and important factors to be considered:

1. **Position of the neonate and team members during resuscitation:**
  Possible positions and placement of the neonate during resuscitation were discussed in the MDT and tested in simulation sessions. Concerns from different professionals were identified and elaborated on in simulation training sessions: time needed to set up the bed for resuscitation, soiling of bed and linen by amniotic fluid and blood, bed mattress’ ability to provide a firm surface for resuscitation, and prevention of infant hypothermia.
2. **Resuscitation equipment:**
  The conventional equipment for resuscitation was added to a mobile stand in collaboration with the local Medical Technical Equipment (MTE) department. Initially, a T-piece resuscitator, suction device, bag and mask, an air-oxygen blender as well as access to hosing for oxygen and air were added. Upon testing, several improvements were made or recommended: These included adding an Apgar timer and a pulse oximeter.
3. **Data collection tools:**
  The MDT group developed data collection tools aiming to ensure collection of important data with minimal increase in workload on the midwives. The data collection tools included three case report forms (CRF) for collecting patient-level data, 1) a documentation sheet from the labor department for collecting birth details, 2) a form for documenting resuscitation efforts and neonatal data, and 3) a form for collecting data in case of admission to the neonatal unit. The tools were developed and tested in a closed feedback loop and were tested on a small scale at the labor department in Malmö.
4. **Training and education:**
  Seminars on the SAVE-study and cord clamping were set up for staff in the labor and neonatal units. Repeated drop-in sessions were held to go through the method for “in-bed” resuscitation with focus on placement, positioning, and equipment. Participants could provide feedback and suggest improvement possibilities.
5. **Communication and recruitment:**

The study set up was communicated to the labor department, neonatal unit as well as the local antenatal units (approximately 20 units in Malmö). We preferred to carry out recruitment at the antenatal units, but it was also possible to recruit women at the labor department. A coordinating midwife was given responsibility for being in close contact with the antenatal units and for continuous provision of recruitment posters, patient information and informed consent materials.

**Fig. 2:**
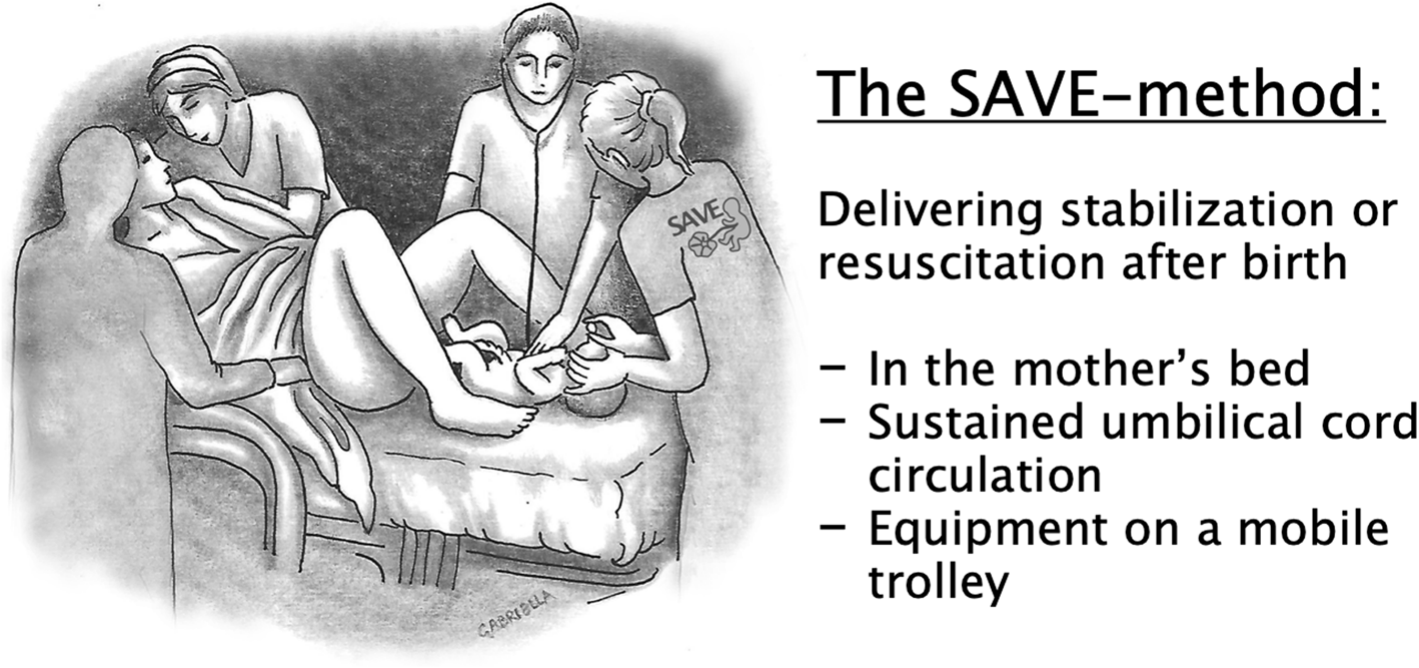
An illustration of ”in-bed” intact cord resuscitation using the SAVE-method

The development of the intervention resulted in the SAVE-method (Fig. 2) which enables ICR to be performed in the mother’s bed, using standard equipment for resuscitation. The foot of the mother’s bed serves as a flat surface for resuscitation and the equipment is provided on a mobile stand equipped with a T-piece resuscitator, suction device, bag and mask, Apgar timer, pulse oximeter and access to oxygen and air.

### Phase II-III: Implementation of the SAVE-method

By adding two other sites in phase II, the study was further developed, carrying out wider scale tests and developing methods tailored to the conditions in different settings. It was possible to use the PDSA cycle process to identify important factors by also utilizing knowledge, feedback, and experiences from phase I and phase II (see Table 1). The process of developing an implementation protocol is further described in the methods section. Methods/Design.

**Table 1:** Important factors identified in the adoption of the SAVE-method to additional sites in phase II

**Introduction phase**	**Approach decision makers and stakeholders** Achieve acceptance for the study from the head of departments and stakeholders (see Table 3)
	**Set up of local team (facilitators)** Form a local team including: a medical responsible doctor, coordinating midwife, obstetrician, neonatologist and neonatal nurse Define responsibilities in the study
**Set-up phase**	**Define setting and develop new local routines** Involve stakeholders to update routines to implement SAVE-protocol eg: • routine for alerting the neonatal team of infants requiring resuscitation • routine for control of SAVE-equipment on mobile stand • routine for moving of the infant from bedside to dedicated resuscitation area if in need of more advanced resuscitation efforts Prepare a checklist in the labor department for implementing the study and planning the birth according to the randomization instruction
	**Set up of local equipment** Establish collaboration with the MTE department to set-up the equipment and adopt the local setting in the room eg. oxygen/air outlets and longer gas hoses
**Start-up phase**	**Set up training sessions for staff (adopters)** Set up simulating exercises including all staff present in the labor department Plan training days with seminars on cord-clamping and SAVE-study process for different groups of staff Develop training material for the introduction of additional sites

### Participants

#### Clinical intervention

##### Inclusion criteria neonates:

- Late preterm and term neonates with gestational age ≥ 35 + 0 in need of resuscitation

##### Exclusion criteria neonates:

- Delivered by cesarean section

Complications at birth that intervene with cord circulation or management of resuscitation e.g. placenta abruption, damage to umbilical cord or congenital malformation of airway or lungs

The intervention group will receive initial resuscitation with an intact cord in the mother’s bed and cord clamping and cutting after 3 min. If the need for ventilation ends, the cord can be kept intact for an additional period. In the control group, the umbilical cord will be clamped and cut directly after birth to allow for ventilation within one minute according to current guidelines.

##### Consent process

Since information and consent cannot be given in the emergency situation, pregnant women and their partners, in the regions participating in the study, are provided information regarding the study at the antenatal departments and preferably asked for consent prior to arriving at the labor department. Otherwise, women and their partners are informed and asked for consent upon arriving at the prenatal or labor department.

#### Implementation development and evaluation

##### Inclusions criteria:

- Managers and medical responsible doctors in the neonatology and labor departments where the SAVE-study is conducted.
- All midwives, auxiliary nurses, obstetricians, pediatric nurses, neonatologists as well as residents and fellows in obstetrics and gynecology, pediatrics and neonatology where the SAVE-study is conducted.
- Parents of included neonates

##### Exclusions criteria staff:

- Staff on long-term absence (e.g. parental or sick leave) will be excluded.

The study protocol was developed within the i-PARIHS determinant framework [22] and the implementation-outcomes evaluation framework [19] will be used for study evaluation. Lessons learned from the PDSA-cycles in phase I and II of the SAVE-study were used to create an implementation strategy for the phase III multicenter trial. The steps used to develop the implementation study protocol are described below.

##### Implementation mapping

In preparation for carrying out phase III, we used what we learned in phase I and II to create the implementation protocol (see Table 2) [23]. We made a needs and assets assessment as an important first step in the implementation mapping process (see Table 2). The level of outcome measurements will be at the individual level of clinical decision makers, adopters and end-users.

**Table 2:** Implementation mapping in the SAVE-study

Implementation mapping steps	Application in the SAVE-study
1.Conduct a needs and assets assessment and identify adopters and implementers	• Review of lessons learned in the process of the stepwise start-up of the SAVE-study, from phase I-III • Identify a feasibility process for start-up of new sites
2.Identify adoption and implementation outcomes, performance objectives, and determinants, create matrice of change	• Identify roles and responsibilities for clinic decision makers, stakeholders, facilitators (the local SAVE-team), adopters and end-users (see Table 3) • Identify implementation outcomes for managers, adopters and parents experiencing the SAVE-method (see Table 3) • Defining determinants of the implementation (see Fig. 3)
3.Choose theoretical models, select or create implementation strategies	• Selection of the PARIHS framework and the implementation outcomes evaluation framework • Development of the logic model of the SAVE-study (Fig. 3)
4.Produce implementation protocol and materials	• Develop data collection aids, instruction videos, CEPS-simulation exercises and lectures to train involved staff • Setting up a multidisciplinary implementation group (MIG) and develop the implementation protocol • Arrange workshops using an adoption of the Delphi model to identify questionnaires to be used when evaluating implementation outcomes • Testing of implementation toolkit when starting up additional sites
5.Evaluate implementation outcomes	• Plan evaluation of clinical and implementation outcomes

**Table 3:** Needs and assets assessment to prepare for implementation of intact cord resuscitation using the SAVE-method (Implementation Mapping Task 1)

**Intervention:** Intact cord resuscitation using the SAVE-method **Setting:** Regional hospital level			
**Target: Role**	**Function**	**Adoption, Implementation and Maintenance Outcomes**	**Performance objectives**
Clinical decision maker	Head of department Head of labor department Head of neonatal unit	The management team at the labor department clarifies economic responsibility and decides to adopt to the SAVE-study protocol by signing the resource agreement	1. Agree to participate in the SAVE-study 2. Gain support from local stakeholders 3. Provide a coordinating midwife and ambassador 4. Answer surveys on implementation outcomes
Stakeholders	Medical managing obstetrician Medical managing neonatologist CEPS instructors Medical technology department	The medical managing obstetrician and neonatologist as well as CEPS-instructors participate in discussions on preparations to be made and local adoptions required for study set-up A dialogue with the medical technology department is needed to prepare facilities and equipment for the SAVE-method	1. Plan and prepare the facilities and equipment 2. Identify practical barriers for implementation 3. Discuss and plan simulation training sessions for staff involved in resuscitation 4. Setting up for study start-up activities 5. Answer surveys on implementation outcomes
Implementer – facilitators	Local SAVE-team: principal investigator (medically responsible), coordinating midwife, responsible for equipment and responsible for data entry	A local principal investigator is identified, and a local SAVE-team is set up. The local SAVE-team participates in the training sessions provided by the central study team, they allot time to prepare for study start-up at the local site and follow-up the study participation continuously. Communicate if any problems arise during the study period	1. Study start-up activities including setting up equipment in collaboration with medical technician, organizing training sessions and simulations for staff 2. Communicate and prepare staff in labor, neonatal and intensive care units 3. Go through local routines and update if needed to adapt to SAVE-protocol 4. Follow-up potential barriers to apply the SAVE-method 5. Give feedback to central study team as well as local staff 6. Motivate and engage staff to maintain recruitment 7.Answer surveys on implementation outcomes
Adopters	Staff in labor departments: midwives, obstetricians, auxiliary nurses and residents Staff in neonatal units: nurses, neonatologists, pediatricians, assistants and residents Intensive care units: anesthesiologist (where relevant)	Staff participates in required training sessions provided. The staff follow the randomization process and adhere to study protocol	1. Follow the randomization process 2. Adhere to study protocol 3. Answer surveys on implementation outcomes 4. Participate in interviews
End Users	Patient – neonate Parents	The parents to the neonate consent to participate in the SAVE-study allowing collection of clinical outcomes, relevant birth data to be used and by answering post-natal surveys	1. The included neonate participates in required measurements of clinical data 2. Parents answer surveys on their experiences of resuscitation and participate in interviews

##### i-PARIHS framework

**Fig. 3:**
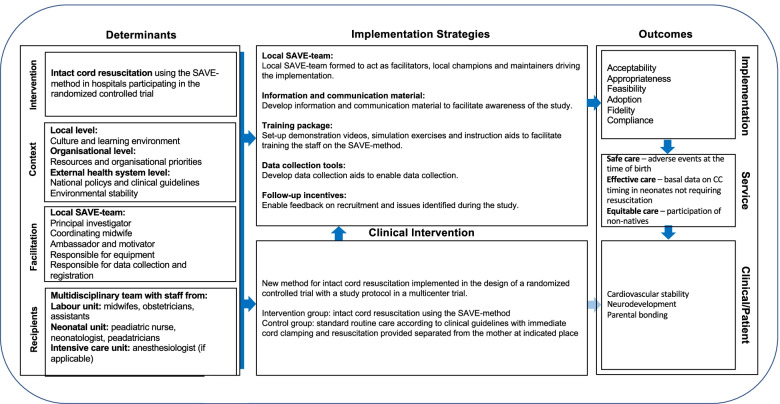
SAVE study logic model developed using implementation mapping moving from pilot study to a multi-center trial

Our findings from phase I and phase II were used to identify determinants that act either as barriers or enablers to implementation of the SAVE-method using the (i-PARIHS) determinant framework [22]. The i-PARIHS framework four core determinants of implementation success (innovation, context, recipients, and facilitation) were used to create a SAVE study logic model (Fig. 3).

The innovation’s role as a determinant comprises both the evidence base behind ICR / the SAVE-method and the method’s feasibility and acceptability. The context is divided into three levels: the regional local level, the organizational level, and the external health care system level [22].

Facilitation as a determinant refers to the local SAVE-team leading implementation and acting as facilitators at each hospital and includes the central project team carrying out the study. The recipients comprise staff in the labor- and neonatal units and the anesthesiologist, where applicable. These people adopt the SAVE-method and the study randomization process, making it part of their practice. The strategies developed and planned for phase III are summarized in the SAVE study logic model (see Fig. 3).

##### Implementation outcomes evaluation framework

The implementation outcome evaluation framework was used to prepare for evaluation in phase III of the SAVE study. The questionnaires were developed using an adaptation of the Delphi process [24]. Three workshops were organized with a multidisciplinary implementation group (MIG) to identify relevant questions for evaluating implementation outcomes in the neonatal resuscitation setting. The MIG comprised members of the neonatal team (pediatrician, neonatologist, neonatal nurse) and the labor team (midwife, obstetrician, head of department representative) and the implementation researcher. As a concluding step, the final questions were graded by the MIG to determine which questions were most relevant and should be included in the questionnaire. The iceberg model in Table 4 shows the research questions and the outcomes and data sources used. The questions chosen, and outcomes evaluated, are shown in Table 4.

**Table 4:** The iceberg model of the organizational culture with study outcomes and data sources defined. The SAVE-method illustrating the practice and the implementation strategy and context illustrating the underlying determinants enabling the implementation of the SAVE-method

	**Questions**	**Outcomes**	**Data sources**
**SAVE-method** **(Intact cord resuscitation)**	Is the SAVE-method clinically effective for the neonate?	Cardiovascular stability and recovery after asphyxia Neurodevelopment after resuscitation Attachment/Bonding patterns after resuscitation (for complete list of clinical outcomes see www.clinicaltrials.gov)	Analysis of clinical routine data Postnatal surveys (ASQ) with parents at 4 and 12 months of age Analysis of clinical data from examination of psycho-motor development at 2 and 5.5 years of age Postnatal surveys on: breastfeeding (BSES) at 2, 4 and 6 months of age parental bonding at 2 and 6 months of age
	How do parents experience the SAVE-method?	Acceptability	Postnatal surveys with parents after birth Individual interviews (qualitative study III and IV)
	What are the attitudes and beliefs regarding cord clamping among different professionals present in the resuscitation situation?	Acceptability	Surveys with staff
	What is current cord-clamping practice in standard care?	Acceptability, Adoption	Surveys with midwives and obstetricians
	What are the experiences of ICR among staff in the labor- and neonatal units?	Acceptability	Implementation surveys with staff Individual interviews (qualitative study I and II)
	Is the SAVE-method applied by staff?	Adoption	Implementation surveys with staff
	What are the barriers and facilitators for using the SAVE-method?	Adoption	Implementation surveys with staff
**Implementation strategies**	Is staff participating in training incentives?	Fidelity of implementation strategy	Implementation surveys with staff
	Is the SAVE-method applied by staff?	Fidelity of the intervention (the SAVE-method)	Analysis of recruitment rate and cord clamping time registered for infants requiring resuscitation
	Is staff adhering to the study protocol?	Fidelity of the intervention (the SAVE-method)	Risk-based monitoring of study database
	Is staff comfortable with the training provided and instructions received?	Fidelity of implementation strategy	Implementation surveys with staff
**Context**	How is the organizational readiness level?	Acceptability	Surveys with staff
	How are resource levels?	Acceptability	Surveys with managers in the labor and neonatal unit
	How are the health service level?	Safety, effectiveness, timeliness, and equity	Analysis of adverse events, cord clamping time and socio-demographics of participants
	Equipment/setting/room	Acceptability	Surveys with managers in the labor and neonatal unit

#### Outcomes

The study will evaluate clinical outcomes of the neonate’s function and symptomatology, as well as the parents’ satisfaction with the resuscitation experience. The implementation outcomes will be evaluated by measuring acceptability, adoption, feasibility, and fidelity. The health service outcomes will be based on IOM Standards of care.

#### Clinical intervention outcomes

The clinical intervention outcomes will be the neonate recovery, morbidity, and long-term neurodevelopment as well as parental bonding and breast-feeding habits. Clinical data will be collected on CRF and then registered in the electronic data capture (EDC) system at each study site. Patient-level data will also be collected from the Swedish pregnancy register, which is a national quality register with data including 92–93% of all patients who experienced labor and 98% of antenatal health care units in Sweden. Data will also be collected from the Swedish neonatal register, which is a national quality register with data from neonatal care units.

All patients for whom the parents have given consent will be assigned a study ID. Allocation to ICR using the SAVE-method or control group with standard care will be decided by randomization shortly before birth.

##### Postnatal survey – parents

**Fig. 4:**
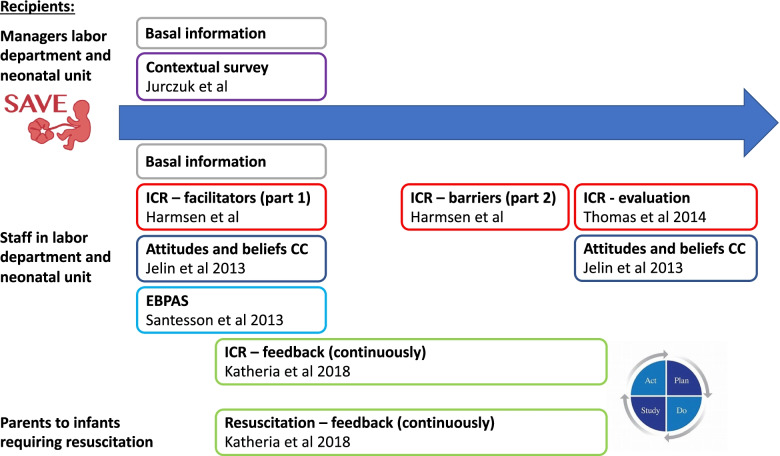
Overview of questionnaires

All parents of infants who have given consent will be invited to answer questionnaires exploring their experience of resuscitation, parental bonding (PBQ) at 2 and 6 months, breastfeeding practice (BSES) at 2, 4 and 6 months of age, and infant neurodevelopment (ASQ) at 4 and 12 months of age. See Fig. 4 for an overview of the questionnaires.

#### Implementation outcomes

Data on implementation outcomes will be collected by electronic surveys as well as qualitative interviews. The surveys will include questionnaires covering identified barriers and facilitators as well as Proctor’s implementation outcomes: Acceptability, Appropriateness, Feasibility, Adoption, and Fidelity (see Fig. 4) [19]. Questionnaires previously used in implementation studies and in studies on delayed CC were identified and adapted by the MIG as described above. The recipients of the intervention are the staff in the labor and neonatal units including midwives, obstetricians, pediatricians, neonatologists, neonatal nurses, and anesthesiologists. Managers in the labor and neonatal units may also help to describe the context. The research questions, outcomes, and data sources are summarized in Table 4.

#### Description of surveys used

##### Context

A background survey including educational level, department and unit, profession, years of experience and research experience will be sent to unit managers and involved staff. A contextual survey will also be sent to unit managers in the labor department and neonatal unit, as well as to medical managing obstetricians and neonatologists to complement subsequent analyses and evaluations of implementation of ICR at each unit. The survey is adapted from Jurczuk et al. [25] and will assess factors identified as significant to implementation of delayed CC. Contextual variables influencing the implementation of delayed CC are resources, level of research activity, equipment, and facilities as well as the impact of the COVID-19 pandemic.

The general acceptability and appropriateness for evidence-based practice at the individual level within different professions present in the resuscitation setting will be measured using the Evidence-based practice attitude scale (EBPAS) [26]. The EBPAS has been translated and validated in a Swedish setting by Santesson et al. [27] and was modified by the MIG to fit in the labor setting. The questionnaire will be sent to staff in the labor department and neonatal unit.

Acceptability and appropriateness regarding cord clamping practice.

Attitudes and beliefs on CC practice among staff involved in the resuscitation setting will be assessed using an adaptation of the questionnaire developed by Jelin et al. [28]. The respondents will be asked to provide general assessments on timing of CC and the use of local documented guidelines on CC management. The routine cord management practice of midwifes and obstetricians will also be assessed to identify barriers of ICR and optimal CC practice in different situations.

##### Adoption

Barriers to and facilitators of ICR will be measured using a modified version of a questionnaire originally developed by Harmsen et al. [29] and translated by Santesson et al. (in manuscript). The questionnaire and will be distributed in two parts. Part one will be distributed at the start of the study to assess completion of training and the need for additional training. Part two will be distributed after 3–6 months of study participation to assess barriers to protocol adherence. This tool will enable creation of a feedback loop to facilitate follow up on concerns or re-training requirements.

##### Usability, acceptability, and feasibility

The usability, acceptability, and feasibility of ICR using a mobile stand (the SAVE-method) will be assessed using questionnaires covering experience of staff involved in the resuscitation as well as parents of infants requiring resuscitation.

Staff experiences will be assessed using an adaptation of the questionnaire developed by Thomas et al. [9] and translated into Norwegian by Sæther et al. [30]. This questionnaire will be used in a feedback loop process and be sent to the staff that has experienced the ICR procedure. An adaptation of the questionnaire will also be used at the end of the study to assess the usability and acceptability on a general level and will be given to all relevant staff in the labor and neonatal units.

Parents’ experiences of the resuscitation will be assessed using an adaptation of the questionnaire developed by Katheria et al. [13]. The questionnaire asks parents about their sense of involvement, information and how comfortable they were during the resuscitation moment.

##### Fidelity

Fidelity will be measured at two levels, the fidelity of the SAVE-method intervention, and the fidelity of the implementation strategy used. The degree to which the study protocol was implemented as intended will be measured by using administrative data following recruitment, protocol adherence and protocol deviations, as well as questionnaires on self-reported training compliance and level of participation in the SAVE-method. Data quality will also be measured by continuous monitoring of the data and feedback to sites as part of the implementation strategy.

#### Qualitative interviews

Qualitative interviews will be carried out in four different study groups. The qualitative studies have a separate ethical approval (EPM: dnr 2021–03,688). All interviews will be conducted via zoom or by live face-to-face approach, recorded and transcribed verbatim to text. The objective of the below interviews is to describe staff and parents’ experiences of resuscitation with an intact cord.

I. **Interviews with staff at labor departments**
  We will conduct 20 interviews with obstetricians, midwives, and auxiliary nurses at the labor department.
II. **Interviews with staff at neonatal units**
  We will conduct 20 interviews with physicians, pediatric nurses, nurses, and auxiliary nurses at neonatal units. The neonatal units in Sweden are divided into three levels of care. We include neonatal units from each level in this study [31].
III. **Interviews with parents**
  We will conduct 20-25 interviews with parents who have been present when their infant needed resuscitation with an intact cord. The parents have given their consent to the study at the antenatal care.
IV. **Interviews with parents when language barriers exist**

When parents with language barriers meet a new healthcare system, it could be difficult to take part in procedures using new methods in the labor department. We will conduct 20 interviews with Arabic-speaking families because it is a common language in Sweden and is spoken in very many countries worldwide. Parents receive information about the study in their own language, but they may not have an interpreter at the labor department and the experience of resuscitation with intact cord can be difficult and misunderstandings can easily arise.

### Health service outcomes

While monitoring and analyzing clinical and implementation outcomes, we will also be able to follow up on health service outcomes in terms of safety, effectiveness, equity, and timeliness.

## Data management

The data from the clinical trial are entered in the EDC system by a designated person at each study site who, by using a secure password, only has access to site-specific patients. The EDC system is stored on a secure server and only named individuals from the project team (monitors) will have access to all sites to enable risk-based monitoring to ensure the quality of the data and to follow-up on recruitment at each site. Prior to analysis, the data from each unit will be cleaned and re-coded to ensure consistent definitions for all variables. Data quality will be assessed by continuous risk-based monitoring and data completeness.

For the implementation evaluation, the questionnaires to parents collecting data on neurodevelopment, breastfeeding, parental bonding, and experience of resuscitation will be sent through the EDC system as electronic surveys. Additionally, the surveys collecting data on implementation outcomes from professionals will also be sent as electronic surveys from a separate part of the EDC system.

### Quantitative data analysis

The data will be summarized and described in the respective treatment groups. The standards for reporting of implementation studies will be used [32]. The implementation process will be described by explaining the characteristics of the context and identifying facilitators and barriers.

Differences between groups will be analyzed with 2-sided tests with 5% significance level. Group comparison between categorical variables will be performed by Chi^2^ test. Group-comparing analyzes of continuous effect variables will be done with Student's T or ANOVA test in normally distributed variables and with Mann–Whitney U or Kruskal–Wallis test in non-normally distributed variables as well as in ordinal variables. Sensitivity analysis will be performed including gestational age, sex and on background variables. Regression models will be built according to sensitivity analysis results.

### Qualitative data analysis

Analysis of qualitative data from study I and II will be analyzed using a thematic analysis with an inductive approach by Clarke and Braun [33]. The data represented in the interviews will go through six non-linear phases; familiarization with the data, coding, searching for themes, reviewing the themes, defining and naming the themes and writing up.

The qualitative data from study III and IV will be analyzed using a phenomenological hermeneutic approach [34]. The interpretation of the text will guide us from comprehension to interpretation, in three phases: naïve reading, structural analysis, and comprehensive understanding.

## Discussion

The strength of this study is the randomized controlled trial design combining the development of a method for ICR, the clinical evaluation of “in-bed” ICR and implementing the method within a multicenter trial. The evaluation of clinical and implementation outcomes may result in an update of the Swedish guidelines and development of an implementation strategy for additional hospitals, which could also be applicable in similar settings throughout the world. The study relies on significant input from the multidisciplinary team and feedback from adopters and parents, covering several perspectives and aspects of conducting ICR in the delivery room. Thereby, the study will provide important knowledge useful for further implementation of methods for intact cord resuscitation. The results will be published in peer-review journals.

## Supplementary Information

**Additionalfile 1**.Guidelines for full-term infants regarding optimal time-point for cordclamping.

## Data Availability

Data sharing not applicable to this article as no datasets has been generated or analyzed yet during the study. After the study is completed, datasets will be available from the corresponding author on reasonable request. Materials and questionnaires from the implementation part of the study are available from the corresponding author on reasonable request.

## Abbreviations

CC: Cord clamping
CEPS: Continuous education of pediatric simulation
EBPAS: Evidence-based practice attitude scale
EDC: Electronic data capture
ICR: Intact cord resuscitation
i-PARIHS: integrated Promoting Action on Research Implementation in Health Services framework
MDT: Multidisciplinary team
MIG: Multidisciplinary implementation group
MTE: Medical Technical Equipment
PDSA: Plan-Do-Study-Act
SAVE: Sustained cord circulation And VEntliation
