# Supplementary material for: A hybrid type I, multi-center randomized controlled trial to study the implementation of a method for Sustained cord circulation And VEntilation (the SAVE-method) of late preterm and term neonates: a study protocol
Source: BMC Pregnancy Childbirth. 2022 Jul 26;22:593. doi: 10.1186/s12884-022-04915-5 (PMC9315331; doi:10.1186/s12884-022-04915-5)
Supplement: Supplementary file 1 — Additionalfile 1.Guidelines for full-term infants regarding optimal time-point for cordclamping. [file 12884_2022_4915_MOESM1_ESM.docx]

| **Additional file 1**. Guidelines for full-term infants regarding optimal time-point for cord clamping. | | | | |
| --- | --- | --- | --- | --- |
| **Guidelines** | **Vaginal birth** | **Vaginal birth requiring resuscitation** | **Cesarean birth** | **Cesarean birth requiring resuscitation** |
| WHO 2014(1) | Clamping not earlier than one minute and up to 3 minutes. | For basic newborn resuscitation, if there is experience in providing effective positive-pressure ventilation without cutting the umbilical cord, ventilation can be initiated before cutting the cord. | The evidence base for recommendations on the optimal timing of umbilical cord clamping for the prevention of postpartum haemorrhage includes both vaginal and caesarean births. The WHO guideline development group considered this recommendation to be equally important for caesarean sections. | For basic newborn resuscitation, if there is experience in providing effective positive-pressure ventilation without cutting the umbilical cord, ventilation can be initiated before cutting the cord. |
| ERC (2) | Where immediate resuscitation or stabilisation is not required, aim to delay clamping the cord for at least 60 s. A longer period may be more beneficial. | Where adequate thermal care and initial resuscitation interventions can be safely undertaken with the cord intact it may be possible to delay clamping whilst performing these interventions. | Not explicitly stated | Not explicitly stated |
| ACOG (3) | Delayed cord clamping at least 30‐60 seconds | Immediate cord clamping | Delayed cord clamping at least 30‐60 seconds | Immediate cord clamping |
| NICE (4) | After administering oxytocin, clamp and cut the cord. Do not cut the cord prior 1 minute, but prior to 5 minutes (if not requested later by the woman, if so support decision). | If the baby is born in poor condition (on the basis of abnormal breathing, heart rate or tone) perform neonatal resuscitation. Cord clamping management not mentioned. | Not explicitly stated | Not explicitly stated |
| Italian guidelines (5) | In term and late preterm newborns, delay cord clamping for at least 60 s and up to 3 min to optimize cardiopulmonary transition and improve iron stores. Consider the mother’s choice if she asks for a longer DCC. Place the newborn on the mother’s abdomen/chest or kept below the perineal plane | Immediate cord clamping | Delay cord clamping for at least 60 s if not cesarean delivery under general anesthesia.  In the case of DCC longer than 60 s ensure the presence of a skilled operator in neonatal resuscitation to evaluate the feto-neonatal transition - Milking the umbilical cord in term and late preterm newborns is a valid option when DCC is not feasible. | Immediate cord clamping |
| Swedish guidelines (6) | Delay umbilical cord clamping between 2-3 minutes | Immediate cord clamping | Delay umbilical cord clamping between 2-3 minutes? Not explicitly stated? | Immediate cord clamping |
| ACOG – The American College of Obstetricians and Gynecologists; ERC – European Resuscitation Council; NICE – National Institute for Health and Care Excellence; WHO – World Health Organization  1. World Health Organization. Guideline: Delayed Umbilical Cord Clamping for Improved Maternal and Infant Health and Nutrition Outcomes. Geneva: World Health Organization; 2014  2. Madar J, Roehr CC, Ainsworth S, Ersdal H, Morley C, Rüdiger M, et al. European Resuscitation Council Guidelines 2021: Newborn resuscitation and support of transition of infants at birth. *Resuscitation* 2021; 161:291-326.  3. Delayed Umbilical Cord Clamping After Birth: ACOG Committee Opinion, Number 814. *Obstetrics & Gynecology* 2020; 136 6:e100-e6.  4. Intrapartum care for healthy women and babies (CG190). *National Institute for Health and Care Excellence: Guidelines* 2017.  5. Ghirardello S, Di Tommaso M, Fiocchi S, Locatelli A, Perrone B, Pratesi S, et al. Italian Recommendations for Placental Transfusion Strategies. *Front Pediatr* 2018; 6:372.  6. Wiklund I, Nordstrom L, Norman M. [Care program for umbilical cord clamping of newborn children]. *Lakartidningen* 2008; 105 45:3208-10. | | | | |
